# External Liver-Derived Complement and Intrinsic Present in Hematopoietic Stem/Progenitor Cells Complosome Modulate Cell Metabolism and Response to Stress

**DOI:** 10.1007/s12015-023-10533-1

**Published:** 2023-03-28

**Authors:** Arjun Thapa, Janina Ratajczak, Magdalena Kucia, Mariusz Z. Ratajczak

**Affiliations:** 1 Stem Cell Program at Division of Hematology, Brown Cancer Center, University of Louisville, 500 S. Floyd Street, Rm. 107, Louisville, KY 40202 USA; 2 Laboratory of Regenerative Medicine, Medical University of Warsaw, Warsaw, Poland

**Keywords:** Complement, Complosome, Innate immunity, Nlrp3 inflammasome, Stem cell homing and engraftment, Stem cell metabolism, Hematopoiesis

## Abstract

Hematopoietic stem/progenitor cells (HSPCs) express receptors for complement cascade (ComC) cleavage fragments C3a and C5a and may respond to inflammation-related cues by sensing pathogen-associated molecular pattern molecules (PAMPs) released by pathogens as well as non-infectious danger associated molecular pattern molecules (DAMPs) or alarmin generated during stress/tissue damage sterile inflammation. To facilitate this HSPCs are equipped with C3a and C5a receptors, C3aR and C5aR, respectively, and express on the outer cell membrane and in cytosol pattern recognition receptors (PPRs) that sense PAMPs and DAMPs. Overall, danger-sensing mechanisms in HSPCs mimic those seen in immune cells, which should not surprise as hematopoiesis and the immune system develop from the same common stem cell precursor. This review will focus on the role of ComC-derived C3a and C5a that trigger nitric oxide synthetase-2 (Nox2) complex to release reactive oxygen species (ROS) that activate important cytosolic PRRs—Nlrp3 inflammasome, which orchestrates responsiveness of HSPCs to stress. Moreover, recent data indicate that in addition to circulating in peripheral blood (PB) activated liver-derived ComC proteins, a similar role plays ComC expressed and intrinsically activated in HSPCs known as “complosome”. We postulate that ComC triggered Nox2-ROS-Nlrp3 inflammasome responses, if they occur within non-toxic to cells' “hormetic range of activation”, positively regulate HSCs migration, metabolism, and proliferation. This sheds a new light on the immune-metabolic regulation of hematopoiesis.

**Graphical Abstract:**

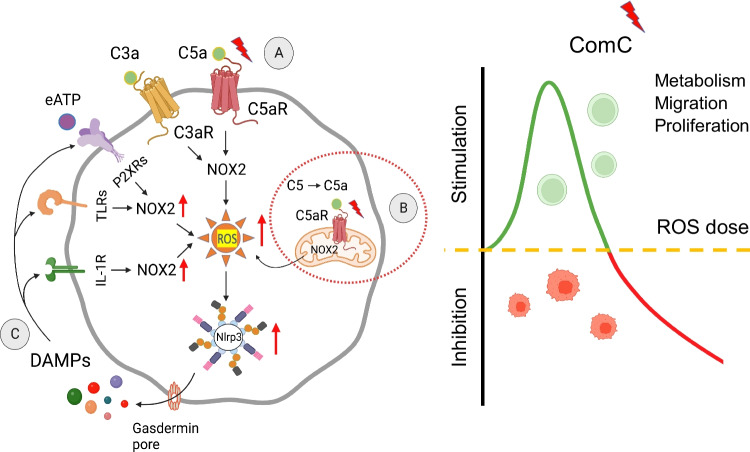

## Introduction

It is well known that hematopoietic cells and lymphocytes share a common stem cell precursor for both hematopoietic and lymphopoietic lineages [1]. While lymphocytes are responsible for acquired immunity, myeloid differentiation of hematopoietic stem/progenitor cells (HSPCs) give rise to phagocytes, mast cells, basophils, eosinophils, and dendritic cells, that are major components of the cellular arm of innate immunity [2]. Lymphocytes and hematopoietic cells also respond to complement cascade (ComC) activation, a central soluble arm of innate immunity [3]. It is known that active ComC cleavage fragments C3a and C5a are strong chemoattractant for innate immunity cells, however they do not directly chemoattract HSPCs. Their effect on migration of these cells is indirect by modulating metabolism and responsiveness to major chemoattractant for these cells including stromal derived factor-1 (SDF-1), sphingosine 1 phosphate (S1P) and extracellular adenosine triphosphate (eATP). This will be discussed later on in this review in context of formation of membrane lipid rafts (MLRs) on HSPCs outer cell membrane.

Mounting evidence accumulated that HSPCs are highly responsive to extrinsic and intrinsic stressors, trauma, and toxic and infectious agents that shape their function and fate. HSPCs respond depending on stressor type, duration, and strength by proliferation and differentiation. To meet these challenges, they need to adjust their metabolism appropriately to adjust demand for energy. In response to external and internal stressors, an important and instructive role also plays bone marrow (BM) microenvironment that modulates HSPCs responsiveness.

ComC is an important regulatory system that senses danger signal-related changes in the cell microenvironment and orchestrates proper cell responsiveness [1–3]. Nevertheless, in addition to activated ComC, there are also other mechanisms that recognize “danger signals” of infectious origin defined as Pathogen Associated Molecular Patterns (PAMPs) or non-infectious Danger Associated Molecular Patterns (DAMPs) [4]. DAMPs are primary mediators of sterile inflammation and are discharged into extracellular space from stressed, damaged, or dying cells [4]. They may be of nuclear-, cytosolic-, mitochondria-, extracellular matrix-, and plasma-membrane origin, and by activating PPRs modulate the proper response of HSPCs to stress.

ComC cleavage fragments C3a and C5a, and DAMPs trigger the state of sterile inflammation in hematopoietic tissues as seen, for example, during mobilization of HSPCs from bone marrow (BM) into peripheral blood (PB) [5, 6] or after conditioning hematopoietic transplant recipient by myeloablative therapy [6, 7]. ComC and DAMPs effects are potentiated by growth factors, cytokines, and chemokines, as well as by non-protein molecules, including extracellular signaling nucleotides, bioactive phosphosphingolipids and eicosanoids. DAMPs family also includes High-mobility group box 1 (HMGB1), S1009A/B proteins, heat-shock proteins (HSP), nuclear and mitochondrial DNA, RNA, and some polysaccharides like for example, hyaluronan fragments [4].

DAMPs, similarly to PAMPs, are sensed by a set of the cell surface and cytosol-expressed pattern recognition receptors (PPRs), including the family of toll-like receptors (TLRs) that are expressed on the outer cell membrane and in the cytosol. Important members of PPRs are also cytosolic NOD-like receptors (NLRs), which recognize both PAMPs and DAMPs [1], and Nlrp3 inflammasome is an important member of the NOD-like receptors family [8]. TLRs and NLRs often regulate cell responses in a coordinated fashion. This is highly relevant to topic of this review because members of both TLRs and NLRs are expressed not only by immune cells but also by HSPCs [3]. As demonstrated, the cooperation between toll-like receptor-4 (TLR4) and NLRP3 inflammasome is involved e.g., in inducing sterile inflammation in BM microenvironment as well as in initiating proper responses of HSPCs in emergency situations [3, 8, 9].

Recent evidence indicates that a state of sterile inflammation is triggered in BM mainly by complement cascade (ComC) cleavage fragments C3a and C5a with the participation of an important mediator of purinergic signaling that is extracellular adenosine triphosphate (eATP) [6, 10]. HSCPs that reside in hematopoietic tissues, to respond appropriately to pro-inflammatory mediators, must adjust their metabolism to meet new challenges. This review will focus on the role of activated C3aR and C5aR and the involvement of some PPRs sensing DAMPs. All these receptors are associated with hematopoietic cell specific nitric oxide synthetase-2 (Nox2) complex that by releasing reactive oxygen species (ROS) triggers Nlrp3 inflammasome for optimal responsiveness to external or internal stressors [11, 12]. We will focus on the biological effects of Nox2-ROS-Nlrp3 inflammasome axis in controlling sterile inflammation in BM and regulating metabolism, and modulating fate of HSPCs. In addition, what is a novel observation is that HSPCs also harbor cytosolic ComC proteins known as “complosome” [13], which can become activated and may modulate as we proposed Nlrp3 inflammasome-mediated cell metabolism and biological functions [12–16].

The role of complosome as the regulator of cell fate has been well described for lymphocytes [12–14], and our recent data indicate its additional regulatory role in normal murine and human HSPCs as well as in BM microenvironment [3, 9, 17]. Therefore, we postulate that HSPCs are not only exposed to liver-derived ComC cleavage fragments, but also their fate is affected by cell expressed intrinsic complosome network [3, 9, 17].

## A novel role for reactive oxygen species (ROS) as signaling molecules in regulating HSPCs metabolism and behavior

Several pathways initiate sterile inflammation in BM that directly or indirectly affect HSPCs. Our team since several years focused on the involvement of activated complement cascade (ComC) C3a and C5a cleavage fragments [5–7], as well as on the effects of purinergic signaling by released in hematopoietic tissues extracellular adenosine triphosphate (eATP) [6]. Both these pathways are activated due to changes in the cell microenvironment surrounding HSPCs, and their soluble mediators interact directly with specific receptors expressed on the outer cell membranes of HSPCs [3, 18].

While, activation of ComC leads to the release of C3 and C5 cleavage fragments—C3a and C5a anaphylatoxins that activate specific G-protein coupled receptors C3aR and C5aR, respectively, eATP released into extracellular space becomes a potent signaling mediator that activates ion-gated purinergic receptors from P2X family—P2X7 and P2X4 [19]. Receptors for C3a, C5a, and eATP are coupled to a superoxide-generating enzyme Nox2 complex that produces reactive oxygen species (ROS) [11], a potent activator of an intracellular PRR that is Nlrp3 inflammasome [12].

However, C3aR and C5aR do not belong to the classical PPRs family; activation of these receptors increases the intracellular level of ROS due to the translocation of cytosolic subunits of Nox2 to the plasma membrane where the Nox2 complex is assembled, facilitates electron transport and produces ROS [11]. In addition, activation of Nox2 complex and release of ROS also occurs after stimulation of several other cell surface PPRs that recognize DAMPs during sterile inflammation, such as eATP, HMGB-1, or IL-1 β [4]. All these signaling pathways potentiates ROS-mediated activation of Nlrp3 inflammasome. In addition, Nlrp3 inflammasome may be additionally activated by cytosolic changes in concentration of K^+^ and Ca^2+^ as seen after stimulation of P2X7 and P2X4 receptors by eATP [19, 20].

As has been postulated, ROS being a product of Nox2, is implicated to be an important signaling molecule in several biological processes in the cells [11, 21]. Pending its activation level, ROS could be beneficial or detrimental for the cells. While a low level of ROS positively affects cell metabolism, survival and proliferation, a higher concentration leads to cell damage due to irreversible damage of structural components, including chromosomal DNA and structural proteins [11]. In contrast, ROS at low concentrations become identified as signaling molecules that can oxidize cysteine and methionine residues in several proteins, that are, enzymes, transcription factors, adhesion molecules, and DNA-associated proteins [11, 22]. These ROS-mediated modifications alter their biological activity and viability. To support this notion, ROS-mediated “redox signaling” controls cell function by modifying the expression and activity of several transcription factors, including for example AKT kinases, NRF2, HIF-1a, FOXOs, HIF-1a, AP1, PTEN, and SIRT1 [11, 22]. Changes in signaling of these transcription factors may affect expression of several intracellular and extracellular enzymes. On the other hand, enzymes themselves could be directly modified by ROS – e.g., those involved in cell metabolism. Interestingly, cell surface expressed CD39 and CD73 ectonucleotidases, that are involved in metabolism of eATP to adenosine are also modulated by ROS [11].

**Fig. 1:**
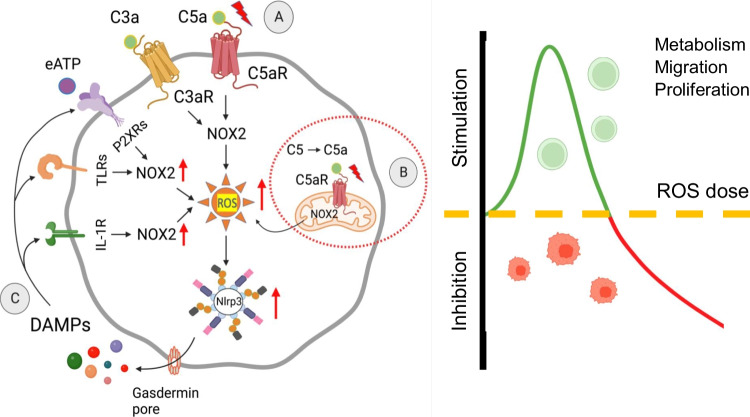
The novel role of Nox2-ROS-Nlrp3 inflammasome axis in regulating the biology of HSPCs. Intracellular ROS in HSPCs is generated by hematopoietic cells specific Nox-2 in response to stimulation by peripheral blood-derived C3a or C5a activating C3aR and C5aR on the outer cell membrane (**A**), and C5aR expressed on mitochondria membrane by complosome-derived intracellular C5a (**B**). Subsequently, generated in cytosol ROS activate Nlrp3 inflammasome that releases several DAMPs or alarmin including IL-1β1, S100A8/A9, HMGB-1 and eATP that interact with corresponding receptors on cell surface to produce more ROS to further augment Nlrp3 inflammasome activation (**C**). These signals within non-toxic to the cell “hormetic zone of activation” [23, 24] enhance metabolism and migration of HSPCs

As mentioned above, ROS activates in cytosol directly Nlrp3 inflammasome that triggers caspase-1 to produce active forms of pro-inflammatory cytokines – interleukin-1 beta (IL-1β) and interleukin 18 (IL-18) that are released from the cells as important DAMPs [4, 20]. Activation of Nlrp3 inflammasome also induces via caspase-1 formation of N-gasdermin pores in the outer cell membrane. These pores are involved in releasing more other DAMPs from the cells, including—HMGB-1, S1009A/B, and eATP [4, 15, 17]. As a result of this Nlrp3 inflammasome activates via the release of DAMPs several specific PPRs expressed on the cell outer membrane, including IL-1R, TLR4, and P2X4/7, that are associated with Nox2 complex and thus amplify further generation of more ROS as depicted at Fig. 1.

## Activation of intracellular metabolic pathways in HSPCs upon activation of Nox2-ROS-Nlrp3 inflammasome axis

As mentioned above, activation of Nox2 complex occurs upon stimulation of cell receptors by cytokines, chemokines, growth factors, as well as what is relevant to this review, also in response to pro-inflammatory mediators. This explains recent research which demonstrated that during inflammation, ComC fragments C3a and C5a or eATP regulate via Nox2-ROS-Nlrp3 inflammasome axis metabolism of HSPCs [16, 17, 21, 22].

To explain this at the molecular level, Nox2-induced ROS “redox signaling” modulates the expression of several enzymes involved in the metabolism of lipids, amino-acid, and glucose [11, 17, 25, 26]. Accordingly, we noticed that stimulation of purified murine Sca-1^+^c-Kit^+^ Lin^−^ (SKL) cells enriched in HSPCs with C3a, C5a, or eATP increases expression of mRNA level for key enzymes involved in the synthesis of cholesterol, including sterol regulatory element-binding protein 2 (SREBP2), 3-hydroxy-3-methyl-glutaryl-coenzyme A reductase (HMGCR), hydroxymethylglutaryl-CoA synthase (HMGCs), and acid sphingomyelinase (ASMAse) [17]. At the same time, we also observed in parallel an increase in the expression of crucial enzymes involved in glycolysis – Glucokinase (GK), Glucose transporter 2 (GLUT2), Phosphofructokinase (PFKFB3), and Glucose-6-phosphate dehydrogenase (G6PD), and mRNA for transmembrane amino acid transporter, large neutral amino acids transporter small subunit 1 (LAT1) [17]. This supports the important role of pro-inflammatory mediators, including C3a, C5a, and eATP in regulating the metabolism of HSPCs during stress and inflammation.

To confirm the role of Nox2-ROS-Nlrp3 inflammasome as mediators in the expression of metabolic enzymes, we exposed cells to small molecular inhibitor of Nlrp3 inflammasome MCC950 and employed as experimental model HSPCs purified from Nox2-KO mice [17]. As expected, inhibition of Nlrp3 inflammasome and lack of proper Nox2 expression in HSPCs significantly affected the expression of all studied metabolic enzymes in response to pro-inflammatory mediators employed in our studies (C3a, C5a, and eATP) [17].

## Proinflammatory mediators regulate migration and metabolism of HSPCs in a membrane lipid raft-depended manner.

**Fig. 2:**
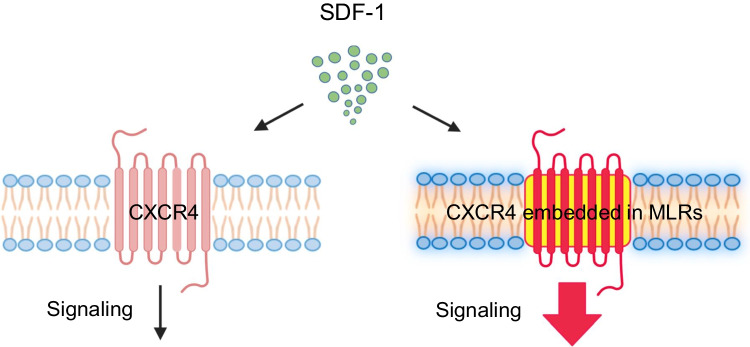
Membrane lipid rafts (MLRs) assemble “raftophilic receptors” with downstream signaling molecules for enhanced biological responsiveness to ligands. It is shown CXCR4 receptor signaling in response to its specific ligand stromal derived factor -1 (SDF-1). Incorporation of CXCR4 into MLRs increases the responsiveness of HSPCs to SDF-1 stimulation. MLRs formation is regulated as we envision by de-novo synthesis of lipid components as well as by assembly of already existing in outer cell membrane and cytosol lipids in response to some “cationic anti-microbial peptides” including e.g., LL-37 cathelicidin or C3a [30, 31]

Mounting evidence accumulated that the formation of membrane lipid rafts (MLRs) on the cell outer membrane has significant regulatory implications for the response of HSPCs to external stimuli [27–29]. Overall, MLRs are nanoscale glycoprotein microdomains ranging from 10–200 nm in size enriched in cholesterol and sphingolipids. Both these lipid compounds depend on the activation of the pentose phosphate cycle that provides NADPH for cholesterol and lipid synthesis. It is well documented that MLRs float freely in the outer cell membrane bilayer and assemble cytosolic signaling molecules with cell surface receptors for growth factors, cytokines, chemokines, bioactive lipids, extracellular signaling nucleotides as well as adhesion molecules [27]. Based on this we can envision, MLRs as some type of sorting hubs for “raftophilic” receptors expressed on the cell surface that enable their optimal signaling. It has been demonstrated that cells much better respond to stimulation by ligands if corresponding receptors are included in MLRs [29]. Therefore, MLRs that are present in outer cell membranes provide a novel level of regulation and integration of microenvironmental signals that translates into biological responses of the cells. This has been depicted in the example of the responsiveness of HSPCs to their major chemoattractant α-chemokine stromal derived factor-1 (SDF-1) (Fig. 2).

As mentioned above, we noticed in our studies that the stimulation of HSCPS by pro-inflammatory mediators increased the expression of several enzymes involved in lipogenesis [17]. As follow-up to studying these enzymes' expression at mRNA level, we performed metabolomic analysis of HSPCs stimulated by hematopoietic growth factors. The most striking difference we observed in our studies in stimulated HSPCs compared to non-stimulated cells was upregulation of metabolites from the pentose phosphate pathway that provides NADPH required for cholesterol and fatty acid synthesis [17, 32]. We noticed upregulation of 19 isoforms of cholesterol isoforms in HSPCs stimulated with KL + IL-3 + TPO compared to unstimulated cells. Of note, among the identified cholesterol compounds upregulated after stimulation cholesterol isoform CE (14:0), also called 1-Myristoyl-cholesterol, is a crucial important lipid component of MLRs. In addition, we identified several other lipid compounds involved in MLRs formation and/or their rebuilding [17]. This data indicates that one of the early changes in stimulated HSPCs is providing lipid compounds that could be utilized in structural changes of outer cell membrane during MLRs formation [17, 18, 27]. This allows cells to better sense and respond to external stimuli. On the other hand, it provides lipids for synthesis of cell membranes in dividing HSPCs.

Finally, the effect of HSPCs stimulation by hematopoietic growth factors and cytokines as well as pro-inflammatory mediators on MLRs formation was confirmed by direct confocal analysis [17]. We noticed that pro-inflammatory factors, including C3a, C5a, and eATP, as well as several hematopoietic growth factors and cytokines increase the assembly of MLRs in HSPCs [8, 27, 29, 33]. This leads, for example as mentioned above and shown in Fig. 2, to inclusion into MLRs of CXCR4 receptor for BM crucial chemoattractant and retention chemokine that is SDF-1 [33]. This has a direct impact on the migration of these cells, as seen for example, in their egress from BM into PB in response to injury or infection to migrate to damaged tissues to play a role in fighting infection by providing locally macrophages and granulocytes [34]. It is also relevant in egress of other types of non-hematopoietic stem cells from BM that play a role in vasculogenesis and organ/tissue regeneration [35]. On the other hand incorporation of CXCR4 receptor into MLRs is important in reverse phenomenon that is homing and engraftment of HSPCs after hematopoietic transplantation [36].

Formation of MLRs is, as we envision, affected by i) de-novo synthesis of lipid components as well as by ii) the incorporation of lipids already present in cytosol, and in outer cell membranes, in response to some “priming agents” that facilitate their assembly. This second mechanism is faster and is promoted, as we described by some small anti-inflammatory cationic peptides such for example, LL-37 fragment of cathelicidin peptide, β2-defensin and circulating in PB C3a [30]. Currently, we are investigating if circulating in PB C5a also plays similarly as C3a in a dual role in MLRs formation, by stimulating the de-novo synthesis of lipid components and their assembling from existing in the outer cell membrane present in cytosol lipid resources.

Activation and migration of HSPCs require energy supply, and as demonstrated, Nox2-ROS-Nlrp3 inflammasome axis in addition to lipogenesis is also involved in the expression of enzymes regulating glycolysis and amino acid transport in stimulated HSPCs [17, 22].

## Intracellular complement network (complosome) as a novel regulator of HSPCs metabolism and fate

For many years scientific community accepted that ComC proteins, including C3 and C5 components, are synthetized exclusively in the liver [1]. However, novel data indicates that C3 and C5 are expressed by normal lymphocytes and other non-hematopoietic cells [12–14]. Moreover, as demonstrated in lymphocytes, C3 and C5 are cleaved/activated inside cells, and C3a and C5a interact with corresponding C3aR and C5aR receptors in an intracrine-dependent manner. This novel regulatory loop operating in lymphocytes has been described as a ‘’complosome’’ being involved in regulating normal T cell immune responses and metabolism [12–14].

To explain this intriguing data, it has been proposed that C3 initially appeared during evolution in ancient single-cell organisms and was involved in the regulation of metabolism [13]. In fact, some of the sequences of this archaic protein have a similarity to enzymes involved in metabolism. With passing time, as evolution progressed, C3 underwent modification into “non-canonical” C3 that, as an intracellular protein, retains some metabolic activities and into “canonical” C3 involved in immune functions as a guardian of pathogen detection and removal [13].

Recently, our team tested the potential role of complosome in regulating the biology of normal HSPCs. To address this question, we isolated from murine bone marrow (BM) Sca-1^+^c-kit^+^lin^−^ (SKL) cells and evaluated by RT-PCR mRNA expression for crucial components of complosome and detected mRNA for C3, C3aR, C5, and C5aR [9, 37]. Next, to elucidate the role of complosome in normal hematopoiesis, we employed as a model C5-KO and C5aR-KO mice [37]. We noticed that these mice in steady-state conditions have a reduced number of SKL cells and clonogenic CFU-GM and BFU-E in BM. Moreover, in Transwell chemotaxis studies, C5-KO and C5aR-KO clonogenic progenitors displayed defective migration to main BM homing chemoattractants, including SDF-1 and eATP. This defective migration of HSPCs has been confirmed in vivo transplant experiments employing BMMNCs isolated from C5-KO and C5aR-KO mice transplanted to WT recipients. We noticed that the number of transplanted PKH26 labeled mutant cells as well clonogenic progenitors, was reduced 24 h after transplantation into lethally irradiated normal recipients as compared to transplanted control WT cells. Similarly, we observed 12 days after transplantation decrease in the number of C5-KO and C5aR-KO cells derived colony forming units in the spleen (CFU-S) and CFU-GM in BM in transplanted normal recipients as compared to transplanted WT cells [37]. This correlated, with defect in Nlrp3 inflammasome activation in murine C5 and C5aR deficient cells. We also evaluated MLRs formation in C5-KO and C5aR-KO HSPCs by confocal microscope, and by Western blot analysis of outer cell membrane fractions, and noticed a defect in mutant cells. This corroborated with a defect in the expression of enzymes involved in lipogenesis, glucogenesis, and aminoacid metabolism [37].

To explain this data, C5aR as part of the complosome network is expressed [12–14] on the mitochondria membrane (Fig. 1). Therefore, stimulation of C5aR by intracrine C5a releases mitochondrial ROS to activate Nlrp3 inflammasome. In normal HSPCs, this step augments, as we postulate intracellular ROS level to enhance Nlrp3 inflammasome activation and leads to additional release of DAMPs. These mediators being released from cells, activate in autocrine-dependent manner their specific receptors associated with Nox2 complex (Fig. 1), and thus additionally increase the intracellular level of ROS that within non-toxic “hormetic zone” potentiates ROS signaling and its metabolic effects [23, 24]. Based on this we postulate that complosome as an intracrine activator of Nox2-ROS-Nlrp3 inflammasome axis is a novel regulator of hematopoiesis. Moreover, this regulatory axis may become a therapeutic target to optimize the proliferation, trafficking, and metabolism of normal HSPCs. Furthermore, it would be necessary in a future studies to assess the role of complosome in regulating the biology of leukemic cells. In support, our unpublished data indicates complosome expression in leukemic cell lines and blasts isolated from leukemia patients.

## Conclusions

We postulate a novel role of liver-derived complement as well as intracellular expressed complement network (complosome) that within safe “hormetic range activation” [23, 24] are important activators of HSPCs metabolism and function. These effects are mediated by triggering Nox2-ROS-Nlrp3 inflammasome axis in response to pro-inflammatory mediators that enhance cell metabolism and biological responses to stress. This data sheds as we propose a new light on the immune-metabolic regulation of hematopoiesis and the responsiveness of HSPCs to stress.

## Data Availability

It is a review paper. No original data have been published.
